# Comparison of Freelite and N-Latex serum free light chain assays: a critical review

**DOI:** 10.11613/BM.2021.030701

**Published:** 2021-08-05

**Authors:** Massimo Daves, Andrea Piccin, Vincenzo Roccaforte, Giuseppe Lippi

**Affiliations:** 1 Clinical Biochemical Laboratory, Hospital of Bolzano, Bolzano, Italy; 2 Northern Ireland Blood Transfusion Service (NIBTS), Belfast, United Kingdom; 3 Department of Internal Medicine V, University of Innsbruck, Innsbruck, Austria; 4 Clinical Pathology Laboratory, Hospital Alessandro Manzoni, Lecco, Italy; 5 Section of Clinical Biochemistry, Verona University of Verona, Verona, Italy

**Keywords:** free light chain, monoclonal gammopathies, analytical techniques and equipment, comparison, immunoassays

## Abstract

**Introduction:**

The measurement of serum free light chain (FLC) represents a fundamental aspect on the assessment of patients with monoclonal gammopathies (MG). Different analytical methods for FLC have become available with the possibility to obtain different value with a substantial impact on the assessment of patients with MG. This study aimed to evaluate FLC results obtained with two different assays and how the difference value obtained can impact in the patient’s assessment.

**Materials and methods:**

Ninety-three patient serum samples that underwent analysis for FLC with two different methods, Serum Freelite (The Binding Site, Birmingham, UK) and N-Latex FLC (Siemens, Marburg, Germany), were included in this retrospective study. Statistical analysis was performed to evaluate correlation, difference, and the grade of concordance between the results obtained with the two methods.

**Results:**

Significant statistical differences between the results obtained from the two methods were found (P < 0.05). A good correlation was found (0.99 for κ FLC, 0.95 for λ FLC, and 0.94 for the κ/λ ratio, respectively). We found a weighted kappa value of 0.65 for κ/λ ratio, 0.65 for λ FLC and 0.90 for κ FLC. A positive bias found with the Bland-Altman plot mirrors overestimation of κ FLC and κ/λ ratio with Freelite compared to N-Latex, whilst a negative bias underscores underestimation of λ FLC by Freelite compared to N-Latex.

**Conclusion:**

Although in general the concordance between Freelite and N-Latex appears satisfactory, several discrepancies could be evidenced and consequently the two assays are not interchangeable.

## Introduction

Monoclonal gammopathies (MG) are characterized by clonal proliferation of plasma cells and the production of monoclonal entire immunoglobulin and/or fragments. Detection and measurement of monoclonal proteins or monoclonal components (MC) are an integral part of diagnosis and clinical follow-up and have been traditionally performed by serum and urine electrophoresis and immunofixation (IFE) (*1*). Human antibodies (Ab) are composed of two identical heavy chains and two identical light chains that are produced in excess of heavy chains, and excess light chains secreted in the blood are called free light chains (*2*). Analytical methods for routine free light chain (FLC) measurement in serum have become commercially available at the beginning of this century so the widespread availability of automated serum assay for measuring and quantifying FLC has generated a significant impact on laboratory assessment of patients with MG (*3*). In 2009 the guideline endorsed by the International Myeloma Working Group (IMWG) recommended the assessment of FLC along with serum electrophoresis and IFE, since these tests were considered the best screening panel for studying plasma cells disorder (*4*).

Now, this testing strategy represents an essential step in the evaluation of MC patients, being capable to detect and measuring light chain MC in almost all patients with non-secreting or oligo-secreting disease and amyloidosis (AL). Furthermore, the measurement of FLC and the derived index, *i.e.,* the ratio between the κ FLC and λ FLC (κ/λ ratio) and involved FLC/non-involved FLC (iFLC/niFLC) ratio, are recommended for the risk stratification of disease progression and in the evaluation of the response to therapy in multiple myeloma or AL (*4*). It has also been suggested that the measurement of FLC may replace in some cases that of Bence Jones protein (BJP). However, in AL as well as in other clinical conditions characterized by elusive MC, urine-IFE must be performed for detecting BJP, such that maximum diagnostic sensitivity can be reached (*5*, *6*). Therefore, this study aimed to evaluate FLC results obtained with a new nephelometer recently commercially available and how the implementation of a different assay for FLC measurement can impact patient assessment.

## Materials and methods

### Study design

This retrospective study was conducted between 1st October 2020 and 30th November 2020 at the Clinical Biochemical Laboratory, Hospital of Bolzano, Italy. The study was planned after implementing the new nephelometer Atellica NEPH 630 (Siemens, Erlangen, Germany) to replace a turbidimeter Optilite (The Binding Site, Birmingham, UK) previously used for the same purposes. All FLC test results performed during the study period were extracted from the laboratory information system (LIS). Serum Freelite (The Binding Site, Birmingham, UK) analyses were measured using the turbidimeter whilst N-Latex FLC (Siemens, Erlangen, Germany) analyses were performed with the new nephelometer. All tests were performed according to the manufacturer’s instructions. This investigation was based on pre-existing data extracted from the LIS in fully anonymised form, so that informed consent was unnecessary. The study was performed in accordance with the Declaration of Helsinki and under the terms of all relevant local legislations.

### Materials and methods

A total number of 93 patients (median age 72 years, range 20-88 years; male 53/93, 0.57) serum samples undergoing routine clinical analysis for FLC at the laboratory were finally included in the study. The concentration of FLC and κ/λ ratio were considered abnormal when results were outside of the reference interval provided by the manufacturers (Freelite κ FLC: 3.3-19.4 mg/L; λ FLC: 5.7-26.3 mg/L; κ/λ ratio: 0.26-1.65; N-Latex FLC: κ FLC: 6.7-22.4 mg/L; λ FLC: 8.3-27.0; κ/λ ratio: 0.31-1.56).

### Statistical analysis

Shapiro-Wilk test for normal distribution showed that all the investigated parameters were non-normally distributed so that results were reported as median, 95% confidence interval (95% CI), and interquartile range (IQR). Wilcoxon’s test was used to assess the statistical significance of differences between the two methods. A P < 0.05 was set as statistically significant. Passing-Bablok regression, Bland-Altman plot, and Spearman’s rank correlation were carried out to analyse the numerical results of κ FLC, λ FLC and FLC-ratio obtained with the two FLC assays. Qualitative concordance of the two FLC assays was explored with the weighted kappa (κ) coefficient, where complete agreement was defined as κ coefficient = 1.00, high agreement as 0.81 ≤ κ coefficient < 1 and a good agreement when 0.61 ≤ κ coefficient < 0.8. The number of patients with iFLC/niFLC ratio ≥ 100 with the two assays was also calculated. Concordance with initial diagnoses was analysed when the κ/λ ratio obtained with the two methods was not concordant. Statistical analysis was done using MedCalc 17.4.4 statistical software (MedCalc Software, Ostend, Belgium).

## Results

**Figure 1:**
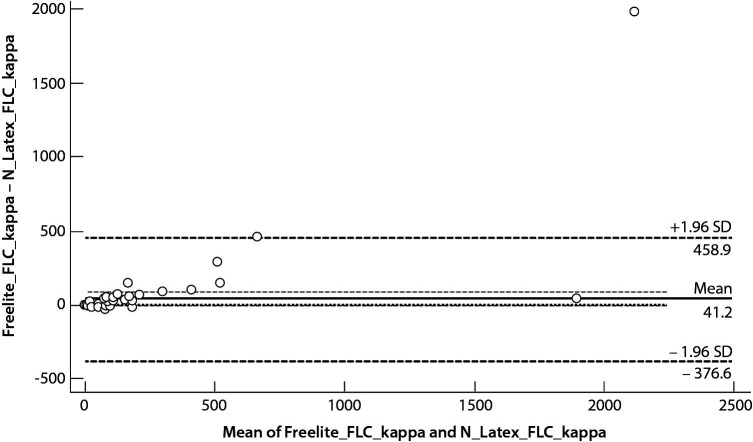
Comparison of N-Latex with Freelite for the determination of FLC kappa using the Bland Altman plot. A positive bias indicates higher values for the determination of FLC by Freelite compared with N Latex FLC. FLC – free light chain. Solid line (mean) – mean difference. Dashed lines (SD) – standard deviation.

**Figure 2:**
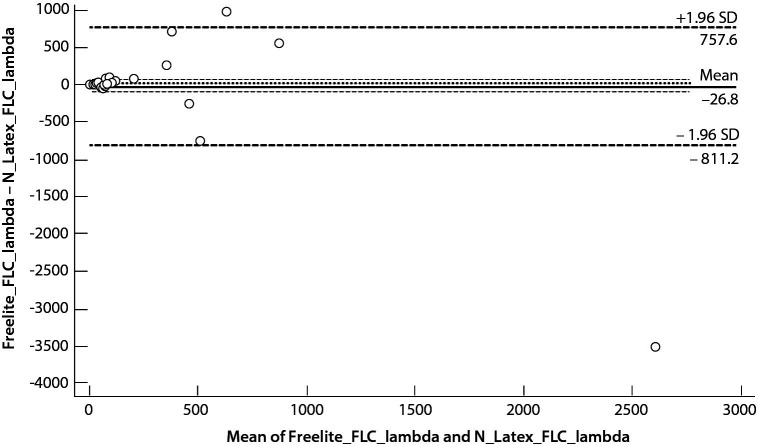
Comparison of N-Latex with Freelite for the determination of FLC lambda using the Bland Altman plot. A negative bias indicates higher values for the determination of FLC by N Latex compared with Freelite FLC. FLC – free light chain. Solid line (mean) – mean difference. Dashed lines (SD) – standard deviation.

**Figure 3:**
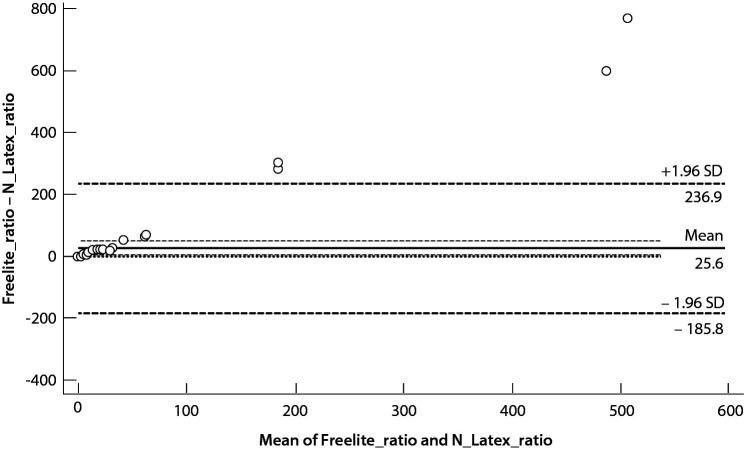
Comparison of N-Latex with Freelite for the determination of FLC κ/λ ratio with Bland Altman plot. A positive bias indicates higher values for the determination of FLC κ/λ ratio by Freelite compared with N Latex FLC. FLC – free light chain. Solid line (mean) – mean difference. Dashed lines (SD) – standard deviation.

The main results of this investigation are shown in Table 1 and Figures 1, 2 and 3. The result of the Passing-Bablok regression analysis for κ FLC is as follows: N-Latex κ FLC = 4.08 (2.97 to 5.01) + 0.78 (0.74 to 0.83) Freelite κ FLC; the Cusum test for linearity showed no significant deviation from linearity (P = 0.130). The result of the Passing and Bablok regression analysis for the λ FLC is as follows: N-Latex λ FLC = 5.42 (3.00 to 7.51) + 1.19 (0.99 to 1.42) Freelite λ FLC; the Cusum test for linearity show no significant deviation from linearity (P = 0.470). The result of the Passing and Bablok regression analysis for the κ/λ ratio is as follows: N-Latex ratio = 0.36 (0.29 to 0.41) + 0.33 (0.29 to 0.36) Freelite ratio; the Cusum test for linearity show no significant deviation from linearity (P = 0.130). Significant statistical differences between values of κ FLC, λ FLC and κ/λ ratio obtained using the two assays were found with Wilcoxon’s test (P = 0.003 for κ FLC, P < 0.001 for λ FLC and P < 0.001 for κ/λ ratio, respectively).

**Table 1:** Comparison of the Freelite and N-Latex FLC methods

	**N Latex**			**Frelite**		
	**sFLC κ** (mg/L)	**sFLC λ** (mg/L)	**κ/λ ratio**	**sFLC κ** (mg/L)	**sFLC λ** (mg/L)	**κ/λ ratio**
Lowest value	1.5	2.2	0.0	1.1	1.3	0.01
Highest value	1870.0	4360.0	188.90	3105.7	1150.2	889.90
Median (95% CI)	32.9 (25.0 - 41.7)	23.2 (19.0 - 30.6)	1.10 (0.98 - 1.21)	31.5 (23.5 - 47.9)	15.0 (10.7 - 18.5)	1.80 (1.42 - 2.19)
IQR	16.1 - 65.0	14.3 - 40.4	0.70 - 2.20	15.3 - 79.3	7.7 - 36.9	1.00 - 6.20
IQR – interquartile range. Cl – confidence interval. sFLC κ – serum free light chain kappa. sFLC λ – serum free light chain lambda.						

**Figure 4:**
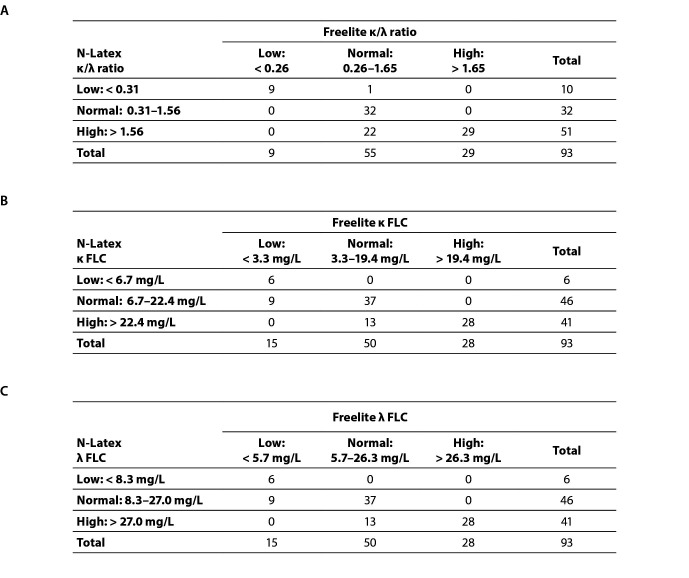
Concordance analysis for the parameters investigated (N = 93). A: FLC κ/λ ratio (Weighted kappa 0.65 (95% CI 0.52 to 0.78). B: κ FLC (Weighted kappa 0.90 (95% CI 0.82 to 0.98). C: λ FLC (Weighted kappa 0.65 (95% CI 0.53 to 0.78). FLC – free light chain.

The Spearman’s rank correlation coefficient was found to be 0.99 (95% CI 0.98 to 0.99) (P < 0.001) for κ FLC, 0.95 (95% CI 0.93 to 0.97) (P < 0.001) for λ FLC, and 0.94 (95% CI 0.91 to 0.96) (P < 0.001) for the κ/λ ratio, respectively. The results of agreement analysis of κ/λ ratio, κ FLC and λ FLC are summarized in Figure 4. We found a weighted kappa value of 0.65 for κ/λ ratio (95% CI 0.52 to 0.78), 0.65 for λ FLC (95% CI 0.53 to 0.78) and 0.90 for κ FLC (95% CI 0.81 to 0.98). The overall number of patients with abnormal κ/λ ratio was 38 (9 patients with ratio < 0.31 and 29 with ratio > 1.56) with the N-Latex compared to 61 (10 patients with ratio < 0.26 and 51 with ratio > 1.65) with Freelite. Only one patient was found to have a pathological low value of κ/λ ratio with Freelite (0.13) and normal value with N-Latex (0.43). This patient especially displayed a large difference in λ FLC concentration, with a high pathological value of 119.2 mg/L measured with Freelite assay compared to only a modest increase (*i.e.,* 39.7 mg/L) with N-Latex, whilst the difference of κ FLC was substantially insignificant (15.6 mg/L and 17.3 mg/L with Freelite and N-Latex, respectively). All patients with abnormally increased κ/λ ratio measured with N-Latex also exhibited abnormal increased with Freelite. Interestingly, 19 patients with increased κ/λ ratio with Freelite displayed normal κ/λ ratio with N-Latex. All these patients displayed a slightly increased value of κ/λ ratio, in a range comprised between 1.67 and 3.37 (median value, 2.16; IQR 1.94-2.44). Bland-Altman plots are shown in Figures 1, 2, and 3. A positive bias reflects overestimation of κ FLC and κ/λ ratio with Freelite compared to N-Latex (Figure 1 and 3), whilst a negative bias underscores underestimation of λ FLC by Freelite compared to N-latex (Figure 2). Finally, a ratio ≥ 100 was found in two patients with both methods, in 3 patients with Freelite and in one patient with N-Latex, respectively.

## Discussion

Taken together, the results of our study reveal a better correlation between the two methods for κ FLC compared to λ FLC and κ/λ ratio. The technical and analytical characteristics of the N-Latex FLC and Freelite assays have been thoughtfully described elsewhere (*7*-*10*). Previous evidence has shown that numerical values produced by the two different systems can have remarkable differences and this aspect has also been evidenced in our study, where higher concentrations are paralleled by an increase in absolute differences (*11*, *12*). Several causes can be preferred for explaining inter-assay variability. First, the Freelite method uses polyclonal Ab, whilst the N-Latex is based on a cocktail of monoclonal Ab. It is hence conceivable that less antigenic determinants may be better recognized by the N-Latex assay, whereas some FLC abnormally expressed in plasma cell dyscrasia could escape detection (*2*, *11*, *12*). Previous data underpins that the discrepancies observed between the two methods may be attributable to polymerization of FLC, leading to potential overestimation using the Freelite method, with concurrent underestimation using the N-Latex assay due to binding sites masking (*13*, *14*). The N- Latex FLC applications on the BN Nephelometer systems have built-in pre-reaction protocols to secure antigen excess protection. Although this protocol seems highly effective for both FLC, samples containing certain monoclonal FLC display a non-linear dilution trend, with a result of the following dilution yielding a higher result than expected (*9*). Furthermore, it is always possible that part of the monoclonal intact immunoglobulin would not be correctly folded, with part of the bound light chain unexposed. If one epitope is exposed, part of the reagent is sequestrated by this bound light chain, thus generating results in false-low FLC concentrations in the initial dilution of the assay (*15*).

Although the vast majority of currently available techniques are based on immunoassays, significant differences in the type of antibodies and in assay design exist. Therefore, their efficiency in coping with the risk of antigen excess, the possibility of missing rare epitopes, the overestimation due to light chain polymerization, the behaviour on serial dilution, and the reproducibility between batches may differ widely. Moreover, due to the lack of an international standard for FLC measurement, it is currently unfeasible to establish whether FLC tests results are underestimated with Freelite or, alternatively, if those obtained with N-Latex are overestimated (*16*).

Overall, our findings demonstrate that the agreement for identifying patients with pathological FLC values seems substantially good for λ FLC and κ/λ ratio, even excellent for κ FLC, though the absolute values differ considerably between the two methods. Similar evidence was provided by previous studies which compared different FLC assays, also revealing significant absolute differences in FLC concentration, especially in samples with high FLC concentrations (*14*-*18*). We also found that the clinical information that can be garnered may differ between these two methods, as we observed 20 patients with altered κ/λ ratio with only Freelite (19 patients with κ/λ ratio above the normal reference range and one below). Our study thus underscores those patients may be differently classified, as being considered at higher risk in case of monoclonal gammopathy of undetermined significance, supposed to have a worse response to treatment in case of MM, or with suspected MG in case of unknown pathological conditions. The iFLC/niFLC ratio is another important information that can be garnered from FLC measurement since a value ≥ 100 is now considered an index of malignancy (*19*). Therefore, this ratio shall be considered an important element in the initial assessment of patients with suspected myeloma. We found six patients with iFLC/niFLC ratio ≥ 100 with at least one assay in our investigation. It is also worthwhile mentioning here that Schieferdecker *et al*. have previously recommended that an iFLC/niFLC ratio ≥ 100 with Freelite may correspond to an iFLC/niFLC ratio ≥ 50 with N-Latex (*11*). By setting the value of 50 with N-Latex as equivalent to 100 with Freelite, the agreement between the two methods was only reached in one such cases, whilst disagreement remained for the other two.

Palladini *et al.* previously compared FLC values measured with Freelite and N-Latex during diagnosis, prognostication, and therapeutic monitoring of AL. The authors concluded that these two assays have similar diagnostic and prognostic performance, though highlighting that they are not interchangeable, so that patient follow-up should be unformed by using a single assay (*20*). Notably, some studies reported κ/λ ratio as a high false positive rate in patients without MG (especially in samples with polyclonal hyper-gamma globulinemia) (*21*). Moreover, a high false negative rate for κ/λ ratio was found in samples with detectable MG (*e.g.,* in the case of lambda light chain monoclonal immunoglobulins) (*22*). These aspects further reinforce the importance of patient monitoring by always using the same assay.

In conclusion, although the concordance between Freelite and N-Latex appears globally satisfactory, several discrepancies could be evidenced. In keeping with previous reports, our findings show that these assays shall not be considered equivalent and are consequently not interchangeable. Caution must be specially used taken with test results of N-Latex for patient classification, considering that the current IMWG guidelines are based on FLC values obtained with Freelite.
